# Biochemical and Biological Evaluation of an L-Asparaginase from Isolated *Escherichia coli* MF-107 as an Anti-Tumor Enzyme on MCF7 Cell Line

**DOI:** 10.52547/ibj.3494

**Published:** 2022-06-13

**Authors:** Masoumeh Shahnazari, Razieh Bigdeli, Aziz Dashbolaghi, Reza Ahangari Cohan, Alireza Shoari, Hossein Hosseini, Davoud Nouri Inanlou, Vahid Asgary

**Affiliations:** 1 Research and Development Laboratory, Javid Biotechnology Institute, Tehran, Iran;; 2 Department of Nanobiotechnology, New Technologies Research Group, Pasteur Institute of Iran, Tehran, Iran;; 3 Molecular and Cellular Biology Laboratory, Department of Quality Control, Pasteur Institute of Iran, Tehran, Iran

**Keywords:** Apoptosis, Breast cancer, Escherichia coli, MCF7 cell line

## Abstract

**Background:**

One of the most widely used anticancer agents is microbial L-ASNase. Herein, we assessed the biochemical and biological properties of an isolated L-ASNase from a Gram-negative bacteria strain, *Escherichia coli *MF-107.

**Methods:**

Using garden asparagus, we obtained several bacterial isolates. These strains were further screened for L-ASNase activity. A promising bacterial isolate was selected for L-ASNase production and subsequent purification. The molecular weight of purified L-ASNase was determined. The MTT assay was applied to assess the cytotoxic effect of the purified enzyme. Also, for caspase activity determination and the apoptotic effect of purified enzyme on in cells, we conducted a real-time PCR method.

**Results:**

The molecular weight of the enzyme was approximately 37 kDa. In the pH range of 7.5 to 8, the enzyme had considerable stability. At 35 °C, the purified L-ASNase optimum activity was recorded. The cytotoxic effect of the enzyme on treated cells was dose-dependent with an IC_50_ value of 5.7 IU/ml. The *Bax *gene expression considerably raised by 5.75-fold (*p *< 0.001) upon L-ASNase treatment. On the other hand, the anti-apoptotic *Bcl-2* gene expression showed a 2.63-fold increase compared to the control (*p *< 0.05). It was detected that the mRNA levels of caspase-3 and p53 were considerably upregulated (5.93 and 1.85-fold, respectively). We did not find any alternation in the caspase-8 activity of the treated cells compared to untreated cells.

**Conclusion:**

In this research, the proliferation of the breast cancer cells remarkably inhibited via the cytotoxic effect of isolated L-ASNase from microbial sources.

## INTRODUCTION

For more than 40 years, L-ASNase (EC 3.5.1.1) has been utilized as a pivotal therapeutic agent against ALL^[^^1^^]^. The L-ASNase is widely found in both eukaryotic and prokaryotic cells and has strongly been investigated over the last decade^[^^2^^]^. 

In the blood vascular system, L-ASNase breaks down L-asparagine to L-ammonia and aspartic acid, thereby restricting necessary exogenous L-asparagine source for leukemic cells. Hence, it can inhibit protein synthesis in these malignant cells, leading to cell apoptosis event^[^^3^^]^. This ability has made L-ASNase an effective anticancer agent^[^^4^^]^. The incapacitation of L-asparagine synthesis via leukemic cells has render the L-ASNase enzyme a valuable drug in antileukemic therapy^[^^5^^]^. Also, an augmented expression of asparagine synthetase in a patient’s primary tumor was most strongly associated with later metastatic relapse^[6]^. The majority of human cells can synthesize L-asparagine from glutamine and aspartic acid; thus, asparagine can be considered a nonessential amino acid^[7]^. Due to lessened asparagine synthetase activity, certain leukemia and other tumors cells, such as sarcoma^[^^8^^]^, lung^[^^9^^]^, breast^[^^10^^]^, and prostate^[^^11^^]^, are incapable to synthesize adequate quantities of asparagine^[12]^. Therefore, based on the dependency on extracellular sources of asparagine, leukemic cells cannot complete protein synthesis^[^^13^^]^. As a consequence of asparagine depletion, L-ASNase elicits a metabolic discrepancy between malignant cells and normal cells to decrease the synthesis of nucleic acids and proteins in leukemic cells^[^^13^^]^. Therefore, L-ASNase can serve as a component of multidrug regimen in adults and children with ALL, which has demonstrated considerable improvement in therapy results contributing to remission in about 90% of patients^[^^13^^]^. It has recently been reported that L-ASNase exhibits antitumor activity not only against leukemia but also toward solid tumors, e.g. lung^[^^14^^]^, cervical^[^^15^^]^, prostate^[^^16^^]^, and colon cancer^[17]^. However, the mechanism of cytotoxic activity of L-ASNase and the determinants of sensitivity to this drug in different cancer types have not been established^[^^18^^]^. Indeed, despite numerous experimental data, the mechanisms underlying the cytotoxic antitumor effects of L-ASNase at the cell level still need to be clarified^[^^19^^]^. This is especially true for basically unknown mechanism(s) by which L-ASNase reduces protein biosynthesis. On the other hand, there is a high demand for novel L-ASNases from new microbial sources that demonstrate less glutaminase activity, ameliorated stability, low Km values, and high substrate affinity to be used as a pharmaceutical drug and achieving the best outcomes in cancer therapy^[^^20^^,^^21^^]^. 

Currently, two types of commercial L-ASNase obtained from *Erwinia chrysanthemi* and *E. coli* are used as chemodrugs^[^^1^^]^. However, these enzymes have some disadvantages such as high glutaminase activity and low substrate specificity, which can cause pancreatitis, neurological seizures, liver dysfunction, leukopenia, and hemorrhages or intracranial thrombosis due to abnormalities in blood coagulation^[^^22^^,^^23^^]^. Thus, it is vital to introduce methods and sources for generating higher contents of glutaminase-free L-ASNases and obtaining greater therapeutic activity and substrate affinity^[^^24^^,^^25^^]^. The aim of this study was to purify and characterize glutaminase-free L-ASNase and evaluate its therapeutic activity in breast cancer cell line. In this research, we described and characterized a bacterial strain in Iran, which produces glutaminase-free L-ASNases.

## MATERIALS AND METHODS

**Bacteria isolation and screening**

Fifteen bacteria species isolated from a garden asparagus were employed in this investigation. For the isolation, the asparaguses were floated in the sterile PBS (DNAbiotech Co. Tehran, Iran) for four hours. Then the PBS was diluted serially and plated on Eosin-Methylene Blue agar (Gibco; Thermo Fisher Scientific Inc.; USA) under sterile condition, whereby single colonies were obtained. Screening for L-ASNase production using the conventional plate assay method with bromothymol blue indicator (Sigma-Aldrich Chemical Co., USA) was performed on the isolated species. The conversion of the color of the medium from green to blue represented the enzyme production^[26]^. Then, the bacterial isolates with positive activity for the production of L-ASNase were selected for quantitative enzymatic assay. 

**Identification of promising isolates via 16S rRNA gene sequence analysis**

From 10-ml cultures grown overnight, DNA of bacterial genomic from the favorable isolates was extracted via DENAzist Asia genomic DNA Isolation Kit (Mashhad, Iran). For the amplification of the 16S rRNA gene, the extracted DNA from each bacterial isolate was utilized as a template using the universal primers 5′ ATC GG(C/T) TAC CTT GTT ACG ACT TC 3′ and 5′ CCA GCA GCC GCG GTA ATA CG 3′. The 25-μL PCR mixtures comprised the following components: 2.5 mM MgCl_2_, 50 mM KCl, 10 mM Tris-HCl (pH 8.3), 1.25 IU of Taq polymerase, 1 μL of DNA template, each dNTP at 0.2 mM, and each primer at 0.2 μM with the final volume brought up to 25 μL with water. PCR was conducted via the following thermocycling program: 10 min of denaturation at 94 °C, followed by 35 cycles of 1 min of denaturation at 94 °C, 1 min annealing at 55 °C, a 2 min of extension at 72°C, and a final extension at 72 °C for 10 min. Afterward, 3 μL of the PCR products were evaluated on a 1% agarose gel made in 1× of Tris/Acetic acid /EDTA buffer and comprising DNA safe Stain. An electrophoresis unit was employed to run the gel at 120 V for 20 min. The migrated bands were detected via UV light and photographed utilizing a gel documentation system. To confirm the existence of properly sized amplicons, the PCR products for each isolate were compared with a 1 kb DNA ladder. The attained sequences for the nominated isolates were aligned and compared with the sequences deposited in GenBank (http://blast.ncbi.nlm.nih.gov/Blast.cgi). A phylogenetic tree was made with MEGA version 5.0 via a neighbor-joining algorithm to define taxonomic position of the isolates.

**Quantitative L-ASNase assay and glutaminase activity determination**

For L-ASNase activity quantification through the method of Imada *et al.*^[27]^, the amount of released ammonia mediated by L-ASNase activity was measured by Nessler’s reaction. Following bacterial culture in the modified Czapek Dox broth medium (NaNO_3_, 0.2 g; KCl, 0.05 g; magnesium glycerophosphate, 0.05 g; FeSO_4_, 0.01 g; sucrose, 3 g; K_2_HPO_4_, 0.035 g; L-asparagine, 0.2 g in 100 ml; Sigma-Aldrich Chemical Co.) for 24 h and centrifugation (10,000 ×g, for 5 min, 4 °C), the reaction was initiated by adding 450 µl of supernatant to 900 µl of substrate mix (50 mM of KCl, 50 mM of Tris-HCl buffer with pH 8, and 20 mM of L-asparagine). For glutaminase activity assay, L-glutamine was employed as the substrate. Incubation was then performed at 37 C for 30 min; the reaction was then stopped by adding 100 µl of 15% trichloroacetic acid (Sigma-Aldrich Chemical Co.). After centrifugation (10,000 ×g, 5 min, 4 °C), the ammonia concentration in supernatant was measured calorimetrically via adding 100 µl of Nessler’s reagent (Fluka; Buchs; Switzerland) to tubes comprising 800 µl of distilled water and 200 µl of supernatant and incubated at room temperature for 10 min. Eventually, using a spectrophotometer (UNICO 2100, Unico Inc., USA) and at the wavelength of 450 nm, the absorbance was read. Ammonium sulfate (Sigma-Aldrich Chemical Co.) was also employed for standard curve preparation where one unit equaled the amount of L-ASNase releasing one micromole of ammonia from L-asparagine per min at 37 °C and pH 8.6.

**L-ASNase production**

Based on a previously described method^[28]^, the selected bacterial strain was grown in 100 ml of the modified Czapek Dox broth medium in an Erlenmeyer flask. Incubation was performed in a controlled environment incubator shaker (WIS-10, South Korea) at 200 ×g and 37 °C for three days. Following centrifugation (10,000 ×g, 20 min, 4 °C) of the culture, the obtained supernatant was used as the enzyme source. Multiple factors affecting ASNase production were improved via changing one factor at a time, namely agitation rate (0.0–300 rpm), temperature (25–50 °C), pH (5.0–8.0), incubation period (12–96 h), carbon sources, and nitrogen sources.

**Protein determination**

To determine the enzyme concentration, the Bradford assay was used^[29]^. For this purpose, the BSA (Bio-Rad Laboratories, USA) was prepared at various concentrations of 1-1000 μg/ml. Thereafter, to 100 µl of each sample, 100 µl of Bradford reagent was added, with incubation being performed for 5 min. Eventually, using a spectrophotometer (UNICO 2100) and at a wavelength of 595 nm, the absorbance was read.

**Protein purification**

The purification was performed on a crude extract based on a modified method explained before^[30]^. To this end, the powdered crude extract was saturated with ammonium sulfate to 70% saturation. Then the mixture was incubated at 4 °C for 12 h. After centrifugation at 4 °C (10,000 ×g, 20 min,), the precipitate was dissolved in a Tris hydrochloride buffer (pH 7.5, 0.01 M) and dialyzed at 4 °C against the same buffer for 12 h. The dialyzed fraction was injected into a Sephadex G-75 column that was pre-equilibrated with the same Tris hydrochloride buffer. The fractions were collected and evaluated for enzyme and protein activity. The highest level of enzyme activity fractions was pooled and lyophilized, and the obtained powder was stored at 4 °C.

**SDS-PAGE**

As formerly described^[31]^, the prepared enzyme was suspended in 100 µl of loading buffer 8% SDS, 200 mM of Tris/HCl (pH 6.8), 5% 2-mercaptoethanol, 0.04% bromophenol blue, and 40% glycerol; Sigma-Aldrich Chemical Co.). The solution was boiled at 100 °C for 5 min and then loaded onto a 15% SDS polyacrylamide gel and run at 120 V. The Coomassie Brilliant Blue G-250 (Sigma-Aldrich Chemical Co.) was utilized for gel staining and visualization of protein bands. 

**Purified ASNase kinetic properties**

To find the ASNase activity optimum pH, a pH scale ranging from 5.0 to 10.0 was tested^[32]^. In this regard, the prepared enzyme was incubated at different pH values for 1 h, and then by the standard assay conditions, the relative activity was determined. Also, by measuring the activity of ASNase at various temperatures, ranging from 25 to 100°C, the enzyme activity optimum temperature was estimated. 

**Cell lines and culture medium**

Procedure in cell culture was performed based on a previously described method^[31]^. Two human cell lines, breast cancer cells (MCF7) and normal human embryonic kidney cells (HEK-293), were acquired from the National Cell Bank of Iran at Pasture Institute of Iran (Tehran). The cells were cultured in DMEM (Gibco) consisting of 1% streptomycin/ penicillin (50 µg/ml and 50 IU/ml, respectively) and 10% FBS (Gibco) and incubated in a humidified atmosphere with 5% CO_2 _at 37 °C. At 80-90% confluence, the cells were detached with trypsin-EDTA (Sigma-Aldrich Chemical Co.) solution.

**Cytotoxicity assay**

The cytotoxic properties of the prepared L-ASNase against breast cancer cell line (MCF7) and normal cell line (HEK-293) were determined using MTT assay^[33]^. To perform this assay, 100 μl of the cell suspension (with a density of approximately 1 × 10^4^ cells/well) was incubated in a 96-well plate at 37 °C for 24 h. Then the cells were treated with enzyme (0.004, 0.012, 0.04, 0.11, 0.33, and 1 IU/ml concentrations) and incubated at 37 °C for 24 h. Next, 100 μl of the MTT solution (0.5 mg/ml; DNAbiotech Co.) was added, and the plate was incubated for 4 h. Finally, the brown formazan crystals were solubilized with 100 μl of isopropanol (Sigma-Aldrich Chemical Co.) with optical density being detected at 570 nm, using a microplate spectrophotometer (UNICO 2100). 

**Apoptotic assay**

***RNA isolation, cDNA synthesis, and qRT-PCR***

Once the cells were treated with L-ASNase for 24 h the total RNA was extracted using the RNeasy Mini Kit (Qiagen Inc., Chatsworth, CA, USA) based on the manufacturer’s instructions. Approximately, 1 µg of total RNA was used in reverse transcription reaction. The mRNA copies numbers of exogenous p53, *Bax*, *Bcl-2*, and the housekeeping gene (GAPDH) were determined by real-time qRT-PCR (Step One, Applied Biosystems, Foster City, CA). Each PCR amplification reaction was conducted in a 20-µl reaction mixture containing 1 µl of each primer (0.2 µM), 10 µl of Power SYBR Green PCR Master Mix (2×), 7 µl of double-distilled water, and 1 µl of cDNA (50 ng). Primer sequences are shown in Table 1. After denaturation at 95 C for 10 min, 40 cycles were followed by 95 C for 30 s and 60 C for 1 min in PCR cycling conditions. By utilizing the comparative threshold cycle, the gene expression levels were determined.

***Caspases activity assay***

To determine the caspase activity, a colorimetric assay was employed (Abnova, Taiwan). For this purpose, IC_50_ concentrations of L-ASN were used to treat cells in 5% CO_2_ at 37 °C for 24 h. Next, the cells were lysed in 50 µl of chilled cell lysis buffer and incubated on ice for 10 minutes. After centrifuging (10,000 ×g, 1 min), the supernatant was moved to a fresh tube, and the protein concentration was measured. Afterwards, for each assay, 50 µl of cell lysis buffer was used to dilute 200-300 µg protein. To each sample, 50 µl of 2× reaction buffer comprising 10 mM of dithiothreitol (Sigma-Aldrich Chemical Co.) was added. Thereafter, 5 µl of the 4 mM of pNA conjugated specific substrates (200 µM of final concentration) was added to each tube individually and incubated at 37 °C for 2 hours. At the end, a MultiSkan plate reader (LabSystems Diagnostic OY, Helsinki, Finland) was used to measure the absorbance of samples at 400 nm^[34]^.

**Table 1:** Primer sequences used in qRT-PCR

Gene	Primer sequence	Product size (bp)
***Bax***	F: 5’-CGGCAACTTCAACTGGGG-3’ R: 5’- TCCAGCCCAACAGCCG-3’	149
***Bcl-2***	F: 5’-GGTGCCGGTTCAGGTACTCA-3’ R: 5’-TTGTGGCCTTCTTTGAGTTCG-3’	114
***P53***	F: 5’-CATCTACAAGCAGTCACAGCACAT-3’ R: 5’-CAACCTCAGGCGGCTCATAG-3’	194
***Caspasae-3***	F: 5’-GCCTGCCGTGGTACAGAACT-3’ R: 5’-GCACAAAGCGACTGGATGAAC-3’	179
***GAPDH***	F: 5’-CCCACTCCTCCACCTTTGAC-3’ R: 5’-CATACCAGGAAATGAGCTTGACAA-3’	75

F, forward; R, reverse

**Fig. 1:**
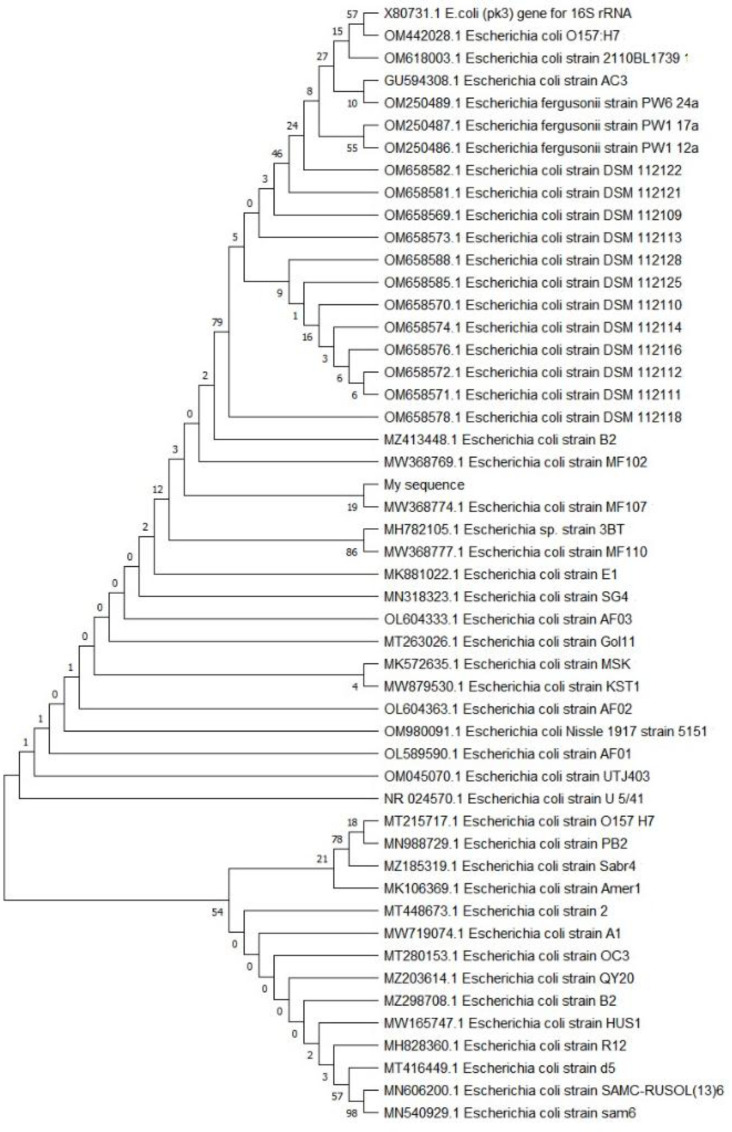
Phylogenetic relationship between the bacterial isolate and other 16S rRNA gene sequences of published strains.

**Statistical analysis**

For statistical evaluating of the experiments, one-way analysis of variance (ANOVA) was utilized. Statistical study was accomplished utilizing SPSS software (version 16.0). Values of *p* lower than 0.05 were predetermined as the criterion of statistical significance.

## Results

**Isolation and screening of bacteria producing ASNase**

In total, 15 bacteria species were isolated from garden asparagus. All bacterial isolates were evaluated for their capability to generate ASNase, utilizing a qualitative rapid plate assay based on bromothymol blue indicator. The color alteration of the medium from green to blue represented the enzyme production. In this step, bacterial isolates, which displayed a remarkable ability to produce ASNase (intense blue zones around the colonies; ≥2 mm), were selected. Morphological properties assessing the colonies demonstrated ASNase generation potential via the plate assay, and one isolate was nominated for further studies.

**Identification of strains via 16S rRNA gene sequencing and phylogenetic analysis**

Amplification and sequencing of the 16S rRNA gene fragment was conducted via the universal primers 27F and 1492R. Via a BLAST search of the National Center for Biotechnology Information databases (https://blast.ncbi.nlm.nih.gov/Blast.cgi?PROGRAM=−blastn&PAGE_TYPE=BlastSearch&LINKLOC=blasthome), the attained sequence data of the16S rRNA gene was compared with the sequences of 16S rRNA regions in the GenBank. Sequences gained from the MACROGEN results and BLAST search, along with an alignment of the 16S rRNA gene sequence of the isolate, displayed that the isolated strain showed highest similarity to *Escherichia coli *MF-107. Multiple sequences taken from the GenBank database were utilized to construct the phylogenetic tree to determine the phylogenetic position of the strain (Fig. 1). 

**Purification of L-ASNase**

**Fig. 2:**
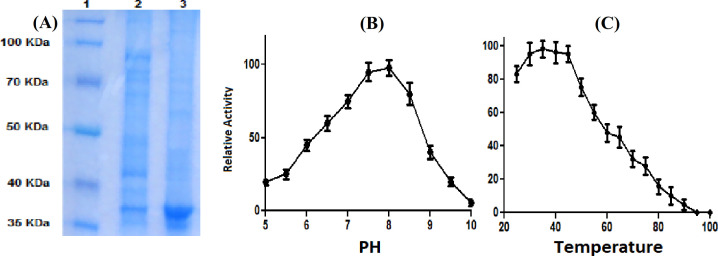
The 15% SDS-polyacrylamide gel electrophoresis of the isolated L-ASNase. Lane 1, protein marker; lane 2, cell-free crude L-ASNase preparation; lane 3, purified L-ASNase (A). Effect of pH (B) and temperature (C) on the activity of L-ASNase. Data are expressed as the mean ± SD of three repeats. The enzyme demonstrated stability within a pH range of 7.5 to 8. The optimum activity of the purified L-ASNasewas recorded at 35 °C.

Ammonium sulfate precipitation and Sephadex G-75 gel filtration methods were utilized for the purification of extracted L-ASNase. The purity and specific activity of the enzyme enhanced with this purification method, while the total activity, total protein, and yield reduced proportionally. A yield of 765 U of ASNase activity and 83.5 mg of protein were acquired for the crude enzyme, with approximately 9.16 U/mg as specific enzyme activity. The purification of the crude enzyme yielded 55.3 mg of protein and 590 U of enzyme activity, with enhanced specific activity of 10.7 U/mg (Table 2). The molecular weight and homogeneity of the purified asparaginase were specified via SDS-PAGE. Our results demonstrated the purity of the L-ASNase preparation compared with the standard molecular weight markers, as only one single distinguished protein band was detected with an obvious molecular weight of about 37 kDa (Fig. 2A). The glutaminase activity assay and SDS-PAGE result revealed that the enzyme purification steps were successful, and the purified enzyme did not show glutaminase activity.

**ASNase stability**

The enzyme activity increased gradually till pH 8, which considered as the optimum pH (Fig. 2B). The enzymatic activity dropped at pH values other than the optimum. The enzyme demonstrated stability within a pH range of 7.5 to 8. The purified L-ASNase optimum activity was observed at 35 °C, which with increasing the temperature, the enzyme activity reduced gradually (Fig. 2C). The enzyme displayed stability within a pH range of 7.5 to 8. The optimum activity of the purified L-ASNase was recorded at 35 °C.

**Table 2:** The activity and concentration of the produced ASNase in the purification procedure

Purification step	Enzyme activity (U)	Protein (mg)	Specific activity (U/mg)	Fold purification	Yield (%)
**Crud extract**	765	83.5	9.16	1	100
**Ammonium sulfate and dialysis**	590	55.3	10.7	1.17	77.12

Fold purification = specific activity (U/mg) of ammonium sulfate and dialysis/specific activity (U/mg) of crud extract. Yield (%) = enzyme activity (U) of ammonium sulfate and dialysis/enzyme activity (U) of crud extract ×100; specific activity = enzyme activity/protein (mg)

**Cytotoxicity assay**

**Fig. 3:**
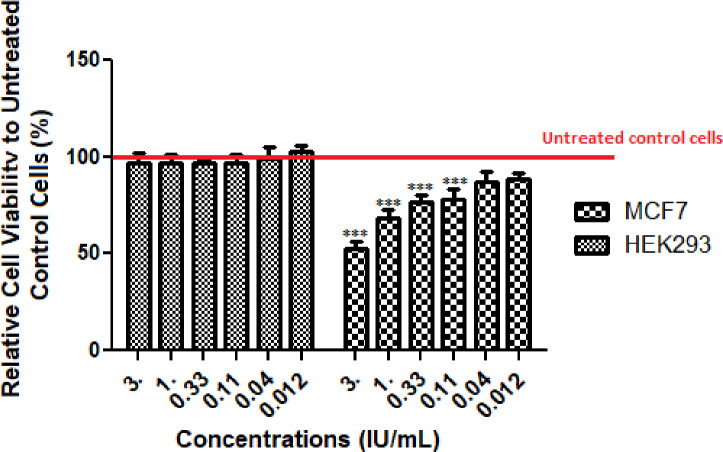
The effect of different concentrations of L-ASNase on the viability of MCF-7 and HEK 293 cell lines by MTT assay. The control group (untreated cells) is equal to 100%. Data are expressed as the mean ± SD of six repeat. Asterisks (^***^) indicates a significant difference with the control group (*p* < 0.001).

For evaluating the cytotoxicity index of the purified enzyme, the MCF7 cell lines were treated with different concentrations of the prepared ASNase for 24 h. The results revealed that the viability of MCF7 cells was affected by asparaginase in a dose-dependent manner (Fig. 3). Viability in MCF7 cells treated with 3, 1, 0.33, and 0.11 IU/ml of the purified enzyme was 52.3, 68.5, 76.6, and 78.1, respectively, and this difference was statistically significant (*p* < 0.001). No significant cytotoxicity was observed at the concentrations of 0.04 and 0.012 IU/ml. Our observations indicated that the effect of asparaginase differs between cell types, and the prepared ASNase enzyme can inhibit the reproduction of cancer cells selectively. However, non-tumor human cell line (HEK-293 cells) was not affected at the concentrations studied. The morphological alterations of the cancer cell line were assessed via an inverted microscope following L-ASNase treatment. The detachment of cells from the surface of the wells demonstrated the cell death. Also, the apoptotic cells represented particular specifications, such as loss of cell adhesion, cellular rounding, membrane blabbing, and shrinkage. The cytotoxic effect of the L-ASNase indicated a remarkable toxic activity (IC_50_ values of 5.7 IU/ml) against MCF7 cells. 

**Fig. 4:**
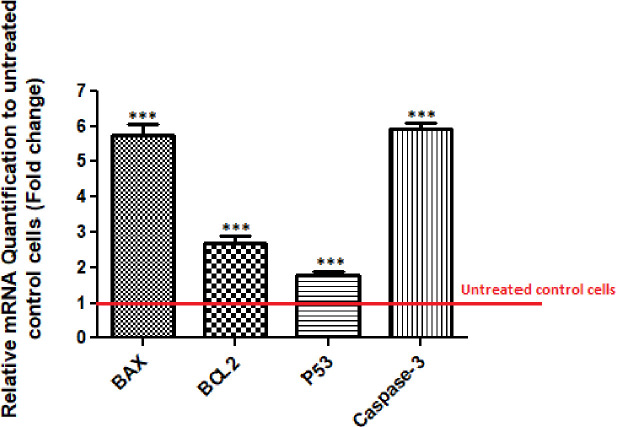
The MCF-7 cells treated with L-ASNase exhibited upregulation at caspase-3, *Bax*, *Bcl-2*, and p53 gene expression levels compared to the control group (untreated cells is equal to 1). Data are expressed as the mean ± SD of three repeats. Asterisks (^***^) indicates a significant difference with the control group (*p* < 0.001).

**Apoptosis-related gene expression evaluation**

The results of qRT-PCR indicated the L-ASNase-mediated upregulation of the *Bax/Bcl-2* ratio. Indeed, the *Bax* gene expression was substantially increased by 5.75 folds in comparison to untreated cells (*p* < 0.001). On the other hand, the anti-apoptotic *Bcl-2* gene expression showed a 2.63-fold increase compared to the control (*p* < 0.05). Also, the considerable up-regulation of caspase-3 and p53 gene expression was detected (5.93 and 1.85-fold respectively; Fig. 4).

**Caspases activity assay**

**Fig. 5:**
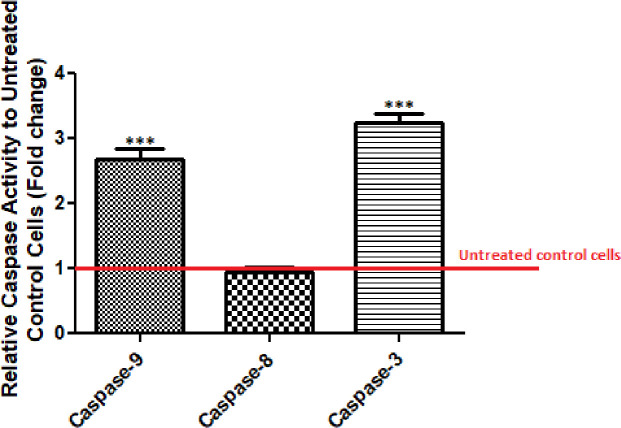
Caspase-3, -8 and -9 activities in MCF-7 cells treated with L-ASNasein comparison to the control group (untreated cells). Asterisks (^***^) indicates a significant difference with the control group (*p* <0.001). Control is equal to 1.

An *in vitro* colorimetric caspases-3, -8, -9 activity assay was conducted to compare apoptosis between the treated cells and control. Our acquired outcomes indicated that L-ASNase considerably enhanced the activity of caspase-3 (3.25-fold) and -9 (2.64-fold), a major apoptotic enzyme, compared to untreated controls (*p* < 0.001). Of note, we did not find any alternation in the caspase-8 activity of the treated compared to untreated cells (Fig. 5).

## DISCUSSION

One of the main attributes of tumors is deficits in the synthesis of one or more amino acids. Also, for protein biosynthesis, tumors cells are intensely dependent on these amino acids from extracellular sources^[35]^. Thus, the deficiency of such amino acid(s) can be considered a valuable opportunity to provide a cancer cell-specific therapy with limited side effects^[36]^. For therapeutic purposes, L-ASNase can be employed to facilitate the degradation of such amino acids, though unwelcome toxicity prevents systemic injection^[37]^. These side effects may occur because in newly synthesized proteins, L-ASNase can hamper multiple forms of glycosylation, including sialylation. Further, glutaminase activity of *E. coli* L-ASNase causes L-glutamine deprivation^[38]^. The common commercially existing bacterial ASNases can lead to toxicity and hypersensitivity during the treatment, highlighting the demand for introducing new sources of these enzymes^[39]^. *In vitro* investigations of L-ASNase effects on different normal and cancer cell lines are essential for identifying involved molecular mechanisms in cancer and developing novel therapy approaches and medications^[40]^. 

In the current study, 15 bacteria species were isolated. For the detection of new bioactive compounds, the asparagus is a valuable source of discovering novel microbial sources. In our research, bacterial isolates displayed the capability to generate ASNase to various extents, and we suppose they can be an encouraging source of anticancer ASNases. Using ammonium sulfate precipitation, the extracted ASNase was purified, followed by dialysis to eliminate extra salt. The specific activity offers a measurement of enzyme purity in the mixture, which is quoted as units/mg. This value represents the enhancement of the purity of an enzyme and a simultaneous reduction in the amount of contaminating protein. The outputs of our study indicated that the purified L-ASNase had the final specific activity of 30.636 U/mg protein, which was purified by 3.33 times.

The transit of various components across the microbial cell membrane and many other processes of enzyme can be affected by the pH of a medium^[32]^. 

Because of particular genetic features of each strain, the most proper pH for ASNase production can be varied^[17]^. In this research, the maximum activity of ASNase occurred gradually with increasing up to pH 8.

SDS-PAGE analysis of the prepared ASNase exhibited one single distinguished protein band with a molecular weight of 37 kDa. The molecular weights of ASNases can differ from each other based on the microbial source of the enzyme. For instance, ASNase from *Streptomyces fradiae*, *Pseudonocardia endophytic* VUK-10, and *Vibrio cholera *ASNase has a molecular weight of ~53^[17]^, 120^[41]^, and ~36.6^[42]^ kDa, respectively. In the present study, we evaluated the purification and glutaminase activity of purified ASNase via glutaminase activity assay and SDS-PAGE, which demonstrated a pure enzyme, free of glutaminase activity. The glutaminase activity of commercially used ASNases is the main concern of the medical community. 

The evaluation of stability and optimal pH of the ASNase in our investigation suggests the potential function in the human body. Stability in physiological pH and temperature is a desirable characteristic for enzymes that are considered as therapeutics. Our data were comparable to several other reported studies^[43-45]^, and the enzyme displayed maximum activity over a wide pH range of 6–9, which was equivalent to values recorded in the human body.

Herein, we assessed the toxicity of the prepared L-ASNase on MCF7 cancer cell lines. The results illustrated that the enzyme was extremely toxic. Comparison of these results with other findings revealed many similarities^[46]^. As an example, L-ASNase from *Bacillus licheniformis* and marine *Bacillus sp.* strains showed a wide cytotoxic effect versus the cancer cell lines such as K-562, MCF7, and Jurkat clone E6-1, with IC_50_ values of 0.153, 0.78, and 0.22 IU, respectively. On the other hand, Farag *et al.*^[25]^ obtained results that were superior to other previously described studies for *Halomonas elongata*, where an L-ASNase enzyme hampered growth in human leukemic cells with significant IC_50_ values (IC_50_ ~1-2 IU/ml). Further, *Bacillus sp.* R36-derived L-ASNase repressed the proliferation of two human cell lines, including colon carcinoma (HCT-116) and hepatocellular carcinoma (Hep-G2) with IC_50_ values of 218.7 μg/ml and 112.19 μg/ml, respectively^[47]^. In addition, asparaginase described by Shafei *et al.*^[48]^ blocked the proliferation of three human carcinoma cell lines, including prostate carcinoma (PC3; IC_50_ ~37 μg/ml), breast carcinoma (MCF7; IC_50_ ~ 12.5 μg/ml), and hepatocellular carcinoma (Hep-G2; IC_50_ ~14 μg/ml). Asparagine can also participate in the Krebs cycle after being converted into oxaloacetic acid and influencing cell metabolism^[49]^. Given that the proliferation of tumor cells such as human leukemic cell lines needs exceeding quantities of these amino acids to generate adequate biomolecules and energy, reductions in the reserve of asparagine will hamper the growth of these cells^[50]^. Furthermore, the study of Karpel-Massler *et al.*^[51]^ displayed the sensitivity of cancer cell lines to the suppression of proliferation via L-ASNase, which lowers the mitochondrial membrane potential and stimulates apoptosis. Muñoz-Pinedo *et al.*^[52]^ concluded that the action of anticancer enzymes can push the cancer cells to starvation conditions as these enzymes convert specific amino acids to an unavailable form for cell usage. Further, Ueno *et al.*^[53]^ found that the depletion of L-asparagine synthetase gene in tumor cells causes the starvation and apoptosis of tumor cells. Also, the lack of asparagine can interrupt the cell cycle.

The outcomes presented herein showed that the cultured MCF7 cell line can be affected by cytotoxic effect of L-ASNase in a dose-dependent manner. The results also revealed that in the presence of L-ASNase, the gene transcription of p53 can be elevated in these cells. Variations in the p53 expression were time-dependent and can be regarded as a mechanism of cell death in response to L-ASNase. 

Our findings indicated the capability of asparagus as a powerful source of bacterial strains for the development of the L-ASNase enzyme. In this research, the isolated L-ASNase from a bacteria strain, *Escherichia coli *MF-107, exhibited a remarkable cytotoxic effect on the multiplication of the breast cancer cells. It was also observed that the enzyme promotes p53-dependent mitochondrial apoptosis pathway. Therefore, it may be a potential candidate with inhibitory impacts on breast cancer cell and also other malignant cells. However, more comprehensive research and studies are required to evaluate this probability, such as half-life determination assays, *in vivo *immunogenicity, pharmacodynamics and pharmacokinetic profiling in animal models, and clinical study on human volunteers.

## DECLARATIONS

### Acknowledgments

The authors are grateful to the people who worked in Javid Biotechnology Institute (Tehran, Iran) for their helpful assistance during the experiment.

### Ethical statement

Not applicable.

### Data availability

The analyzed data sets generated during the study are available from the corresponding author on reasonable request.

### Author contributions

MS: running the project; RB: performed the laboratory assessments; AD: conceptualization; RAC: advisor; AS: writing the manuscript; HH: collected samples and analyzed the data; DNI: supervision and project administration; VA: writing original draft and project management.

### Conflict of interest

None declared.

### Funding/support

This research did not receive any specific grant from funding agencies in the public, commercial, or not-for-profit sectors.
